# Prevalence and clinical relevance of low levels of Pru p 3 specific IgE in patients with peach sensitization

**DOI:** 10.1186/2045-7022-3-S3-P109

**Published:** 2013-07-25

**Authors:** M Pascal, A Mensa, J Sánchez-López, J Milà, L Millan, M Juan, A Valero, R Vilella, C Picado, J Yagüe, J Bartra

**Affiliations:** 1 Immunology, Hospital Clinic, Barcelona, Spain; 2 Unitat Al.lèrgia, Servei de Pneumologia, ICT, Hospital Clínic de Barcelona, Spain

## Background

The peach Lipid Transfer Protein (LTP), Pru p 3, is considered to be a major allergen in peach allergy, but sensitization to it is almost always present in LTP-syndrome. Thus, detection of Pru p 3 specific IgE (sIgE) is frequent in routine peach and/or LTP allergy diagnosis. ImmunoCAP is one of the most frequent immunoassays for specific IgE detection, with a 0.1kU/L detection limit, although 0.35kU/L has traditionally been adopted as the positivity cutoff. The aim was to analyze the prevalence and clinical relevance of low levels of Pru p 3 sIgE in a cohort of patients diagnosed of peach sensitization.

## Methods

Pru p 3 sIgE levels (f420, ImmunoCAP) were analyzed in patients with peach sensitization according to positive skin prick test. Data about clinical symptoms with plant-food, cofactor contribution and plant-foods sensitization were collected on 2 subsets of these patients: below 0.35kU/L (n=33, Group A) and above 17.5kU/L (n=25, Group B).

## Results

319 patients had Pru p 3 sIgE levels above 0.1kU/L. Of these, 42 (13.2%) had values between 0.1-0.35kU/L, 21 (6.6%) between 0.36-0.75kU/L (class 1), 63 (19.8%) between 0.76-3.5kU/L (class 2), 79 (24.8%) between 3.6-17kU/L (class 3), 28 (8.7%) between 17.5-50kU/L (class 4) and 2 (0.6%) between 50.5-100kU/L (class 5).

According to the clinical history of groups A and B, contact urticaria was reported in 9 (27.3%) patients and in 5 (20%), respectively. 16 patients tolerated pulp-peach in A (48.5%) and 9 (36%) in B. Of those with symptoms upon peach ingestion (A: 17 and B: 16), 5 (29.4%) have only presented oral allergy syndrome in A, whereas only 2 (12.5%) in B. The rest had episodes of systemic reactions [(A vs. B in %): urticaria/angioedema (23.5 vs. 31.3%), gastrointestinal disorder (17.6 vs. 56.3%), and anaphylaxis (35.3 vs. 18.8%)]. Patients in B had a broader spectrum of sensitizations to other plant-foods than A and presented more symptoms with plant-foods other than peach. Cofactors, NSAIDs and exercise, were involved in 3 (17.6%) patients in A, whereas in 8 (50%) in B.

## Conclusion

Pru p 3 sIgE below 0.35kU/L should not be regarded as irrelevant in patients with a clinical suspicion of peach and/or LTP allergy. The role of cofactors seems to be relevant in the expression of LTP allergic symptoms, but not to be related to IgE specific levels. IgE levels do not correlate with severity of symptoms, but seem to correlate with a broader spectrum of sensitization to plant-foods non-taxonomically related to peach.

## Disclosure of interest

None declared.

